# Outcomes and Drain Use in Laparoscopic vs. Converted Open Cholecystectomy Cases: A Retrospective Cohort Study

**DOI:** 10.7759/cureus.93233

**Published:** 2025-09-25

**Authors:** Parin Y Patel, Milind K Akhani, Ronak Rathod, Bhavin Baria

**Affiliations:** 1 General Surgery, Health1 Super Speciality Hospital, Ahmedabad, IND; 2 Surgical Gastroenterology, HPB Surgery and Liver Transplantation, Health1 Super Speciality Hospital, Ahmedabad, IND; 3 General Surgery, Shalby Multi-Specialty Hospitals, Ahmedabad, IND

**Keywords:** cholecystectomy, conversion to open surgery, drain placement, gallstone disease, laparoscopic surgery, postoperative complications, surgical outcomes

## Abstract

Introduction and aim: Laparoscopic cholecystectomy (LC) is the gold standard for gallstone disease, but conversion to open surgery may be required in difficult cases. The role of drain placement after cholecystectomy remains controversial. This study aimed to evaluate surgical outcomes of LC, with emphasis on converted cases, and to assess the impact of drains on postoperative morbidity.

Methods: We conducted a retrospective cohort study of 287 patients who underwent LC between January 1, 2023, and December 31, 2024, at a tertiary care hospital. All cases were initiated laparoscopically; conversions were analyzed separately. Data on demographics, comorbidities, operative details, drain use, and postoperative outcomes were collected. Statistical analysis was performed using chi-square or Fisher’s exact test for categorical variables and the Mann-Whitney U test for continuous variables, with p<0.05 considered significant.

Results: Of 287 patients, 272 were eligible for analysis; 223/272 (82.0%) underwent completed LC, and 49/272 (18.0%) required conversion. Converted cases had longer operative times (median, 90 min vs. 60 min; p<0.001), higher complication rates (15/49, 30.6% vs. 25/223, 11.2%; p=0.001), and longer hospital stays (median, seven vs. four days; p<0.001). In completed LC, drains were used in 30/223 (13.5%) patients, while in conversions, 32/49 (65.3%) received drains. Drain placement did not significantly reduce complications in either group but was associated with prolonged hospitalization (five vs. four days in completed LC, p=0.03; eight vs. six days in conversions, p=0.04).

Conclusion: Laparoscopic cholecystectomy was safe and effective, with most procedures completed laparoscopically. Conversion was associated with increased morbidity and longer recovery. Drain placement did not reduce postoperative complications in either completed or converted cases, but was associated with prolonged hospital stay. Selective use should be reserved for technically challenging situations.

## Introduction

Gallstone disease is one of the most common surgical conditions worldwide, affecting 10-15% of adults and representing a major burden on healthcare systems [1]. Cholecystectomy is the definitive treatment for symptomatic cholelithiasis and acute cholecystitis [2]. Since its introduction in the late 1980s, laparoscopic cholecystectomy (LC) has become the standard of care, largely replacing the open approach because of its advantages in terms of reduced pain, faster recovery, lower morbidity, and improved cosmetic outcomes [3,4].

Despite these advantages, LC can be technically challenging, particularly in patients with severe inflammation, adhesions, or distorted biliary anatomy. In such situations, conversion to open cholecystectomy becomes necessary to ensure patient safety and avoid iatrogenic complications [5]. Conversion rates vary between centers, generally ranging from 2% to 15%, and are influenced by patient comorbidities, disease severity, and surgeon experience [6,7]. Although conversion is not considered a complication, it is often associated with longer operative times, increased postoperative morbidity, and prolonged hospital stay compared with completed LC [8].

Another area of ongoing debate in cholecystectomy is the use of intra-abdominal drains. Traditionally, drains were inserted with the expectation of preventing bile collections and detecting postoperative bleeding [9]. However, randomized controlled trials and meta-analyses have shown little evidence to support routine drainage after LC, with studies reporting no significant reduction in complications but a consistent trend toward increased patient discomfort and longer hospitalization [10,11]. According to the most recent World Society of Emergency Surgery (WSES) guidelines, routine prophylactic drain placement after laparoscopic, converted, or open cholecystectomy is not recommended. Instead, selective use is advised only in technically difficult cases, such as bile spillage, uncontrolled bleeding, or subtotal cholecystectomy [12].

In India and many other parts of Asia, LC is widely performed, and drains continue to be used frequently, even in uncomplicated cases [13,14]. However, data examining outcomes in patients who undergo conversion, particularly with respect to drain use, remain limited.

In this context, the present study was undertaken to evaluate the surgical outcomes of patients undergoing laparoscopic cholecystectomy at our institution, with particular emphasis on those requiring conversion to open surgery. A secondary objective was to assess the impact of drain placement on postoperative morbidity, hospital stay, and recovery in both completed LC and converted cases.

## Materials and methods

Study design and setting

This retrospective cohort study was conducted at Health One Super Speciality Hospital, Ahmedabad, India. The study period extended from January 1, 2023, to December 31, 2024.

Study personnel and case allocation

All operations were performed by a single consultant general surgeon with a consistent assisting team. Assistants (residents/OT staff) did not act as primary surgeons. Case allocation was consecutive and determined by clinical presentation and operating-room scheduling (elective list and emergency theatre); there was no preselection or diversion of cases based on anticipated difficulty.

Participants

All patients aged 18 years and above who underwent laparoscopic cholecystectomy (LC) during the study period were included. In our institution, LC is the standard initial approach for all patients with gallbladder disease, and no cases are scheduled as primary open cholecystectomy. Patients who required conversion to open cholecystectomy due to intraoperative difficulty were analyzed as a separate group. Patients undergoing concomitant major hepatobiliary or gastrointestinal procedures (e.g., common bile duct exploration, hepatectomy, pancreatic surgery) and those with incomplete medical records were excluded.

Data collection

Patient data were extracted from electronic hospital records, operative notes, anesthesia charts, and discharge summaries using a standardized proforma. Baseline variables included age, sex, comorbidities (such as diabetes mellitus and hypertension), and American Society of Anesthesiologists (ASA) physical status classification.

Operative details recorded were operative time (minutes), indication for surgery (symptomatic cholelithiasis, acute cholecystitis, empyema, gallbladder perforation), and whether conversion from laparoscopic to open cholecystectomy was required. Intraoperative findings, such as dense adhesions, empyema, gangrenous gallbladder, and bile spillage, were documented. Drain placement was carefully noted, including whether a drain was used, type of drain, and duration of placement.

Postoperative outcomes assessed were surgical site infection (SSI), bile leak, intra-abdominal abscess or collection, hemorrhage, postoperative ileus, pulmonary complications, and in-hospital mortality. Additional variables included length of hospital stay (LOS, in days) and 30-day readmission.

Outcomes

The primary outcome was the overall rate of postoperative complications. Secondary outcomes included LOS, conversion rate, 30-day readmission, reoperation, and mortality. A subgroup analysis was conducted to evaluate the impact of drain placement in both completed LC and converted cases.

Operative technique

A standard four-port laparoscopic cholecystectomy was performed. Strasberg’s critical view of safety (CVS) was required before clipping and division of cystic structures. If CVS could not be achieved or safety was in doubt, the procedure was converted to open cholecystectomy. Conversion triggers were failure to obtain CVS after reasonable attempts, uncontrolled hemorrhage, hostile/obscured Calot’s triangle from dense adhesions or severe inflammation, and intraoperative suspicion of biliary injury. Intraoperative decisions were taken by the consultant operator.

Definitions

Drain placement referred to the use of a closed-suction subhepatic drain. Drains were not used routinely but were inserted selectively at the discretion of the operating surgeon, typically in cases with bile spillage, uncontrolled bleeding, difficult dissection with uncertain hemostasis, or severe inflammation. When used, drains were reassessed clinically; in the absence of bilious or hemorrhagic output and with stable parameters, removal was planned at the first dressing round (typically within 24-48 hours).

Postoperative complications were classified according to the Clavien-Dindo grading system. Minor complications managed conservatively, such as surgical site infection, ileus, or pulmonary events, were considered grade I-II. Complications requiring procedural or surgical intervention (e.g., drainage of intra-abdominal collection) were classified as grade III. Life-threatening complications requiring intensive care (grade IV), while mortality was classified as grade V.

Any complication was defined as the occurrence of at least one postoperative adverse event. This included surgical site infection (SSI, defined as per CDC criteria), bile leak (bilious output from a drain or radiologically confirmed collection), intra-abdominal abscess or collection (requiring radiologic or surgical intervention), postoperative hemorrhage, ileus (failure of bowel function >3 days requiring supportive therapy), or pulmonary complications (pneumonia, atelectasis, or respiratory failure). Mortality was defined as in-hospital or 30-day death from any cause.

Statistical analysis

Data were entered into Microsoft Excel and analyzed using IBM SPSS Statistics version 25.0 (Armonk, NY: IBM Corp.) for Windows. Categorical variables in baseline and operative characteristics were compared using the chi-square test. Postoperative outcomes and subgroup analyses, which included smaller cell counts, were analyzed using Fisher’s exact test. Continuous variables, such as age, operative time, and length of stay, were compared using the Mann-Whitney U test. A two-tailed p<0.05 was considered statistically significant.

## Results

**Figure 1:**
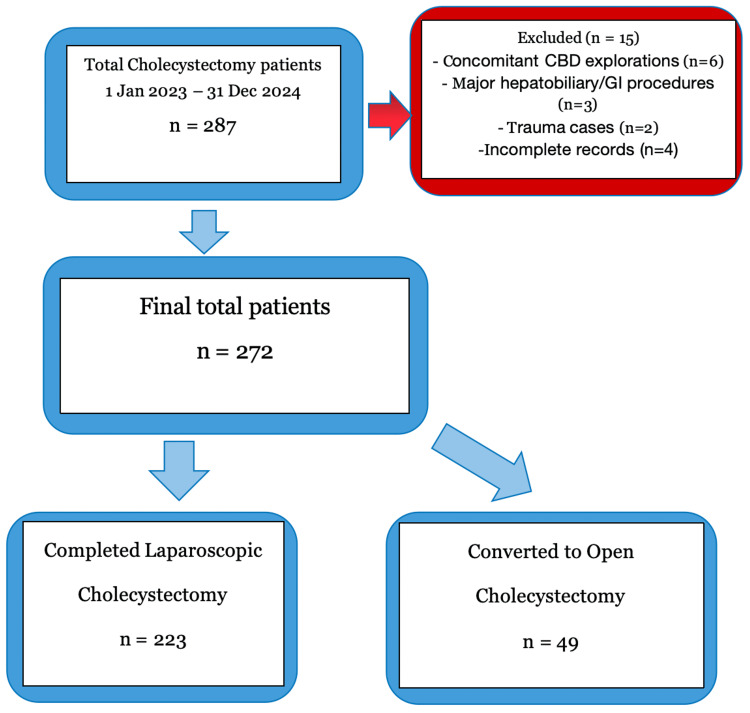
Flow diagram of patient selection. CBD: common bile duct

Figure 1 shows the study cohort. Of 287 patients undergoing cholecystectomy, 15 were excluded due to concomitant procedures, trauma, or incomplete records. The remaining 272 patients were analyzed. Of these, 223/272 (82.0%) were completed laparoscopically, while 49/272 (18.0%) required conversion to open cholecystectomy. The overall conversion rate was 18.0%.

The baseline demographic and clinical characteristics are shown in Table 1. Patients who required conversion were slightly older than those with completed LC (median age: 53 vs. 46 years, p=0.01). The proportion of males was higher in the conversion group (22/49, 44.9% vs. 74/223, 33.2%; p=0.12). Rates of comorbidities, including diabetes and hypertension, were comparable between groups. American Society of Anesthesiologists (ASA) ≥III was more common among converted patients (14/49, 28.6% vs. 38/223, 17.0%; p=0.06). Emergency procedures were significantly more frequent in the conversion group (20/49, 40.8% vs. 33/223, 14.8%; p<0.001).

**Table 1:** Baseline demographic and clinical characteristics of patients undergoing laparoscopic cholecystectomy (LC) and converted cases. P-values derived from Mann-Whitney U test (age) and chi-square test (categorical variables). LC: laparoscopic cholecystectomy; ASA: American Society of Anesthesiologists; IQR: interquartile range; N: total number of patients; n: number of patients

Characteristics	Completed LC (N=223)	Converted (N=49)	p-Value
Age (years), median (IQR)	46 (38-58)	53 (42-65)	0.01
Male sex, n (%)	74 (33.2)	22 (44.9)	0.12
Diabetes, n (%)	62 (27.8)	17 (34.7)	0.34
Hypertension, n (%)	68 (30.5)	16 (32.7)	0.78
ASA ≥III, n (%)	38 (17.0)	14 (28.6)	0.06
Emergency surgery, n (%)	33 (14.8)	20 (40.8)	<0.001

Operative details are presented in Table 2. The median operative time was significantly longer in converted cases compared with completed LC (90 vs. 60 minutes, p<0.001). Indications for surgery included symptomatic gallstones, acute cholecystitis, empyema, and gallbladder perforation. Difficult intraoperative findings such as dense adhesions, empyema, and gangrenous gallbladder were more common in converted cases. Drains were placed in 30/223 (13.5%) of completed LC and 32/49 (65.3%) of converted cases (p<0.001).

**Table 2:** Operative details of completed laparoscopic cholecystectomy and converted cases. P-values derived from Mann-Whitney U test (operative time) and chi-square test (categorical variables). LC: laparoscopic cholecystectomy; IQR: interquartile range; N: total number of patients; n: number of patients

Operative variables	Completed LC (N=223)	Converted (N=49)	p-Value
Operative time (min), median (IQR)	60 (50-70)	90 (80-105)	<0.001
Symptomatic stones, n (%)	148 (66.4)	25 (51.0)	0.04
Acute cholecystitis, n (%)	51 (22.9)	15 (30.6)	0.26
Empyema, n (%)	15 (6.7)	6 (12.2)	0.18
Perforation, n (%)	9 (4.0)	3 (6.1)	0.49
Drain placed, n (%)	30 (13.5)	32 (65.3)	<0.001

Postoperative outcomes are summarized in Table 3. Overall complications occurred in 25/223 (11.2%) of completed LC compared with 15/49 (30.6%) of converted cases (p=0.001). Surgical site infection (SSI) was significantly more frequent in conversions, occurring in 8/49 (16.3%) compared with 12/223 (5.4%) of completed LC (p=0.014). The median length of hospital stay was also longer in converted cases (seven vs. four days, p<0.001). Readmission and mortality rates were low and comparable between groups. Importantly, no bile duct injury (BDI) was identified in either the completed LC or converted groups during the study period.

**Table 3:** Postoperative outcomes of completed laparoscopic cholecystectomy and converted cases. P-values derived from Mann-Whitney U test (LOS) and Fisher’s exact test (categorical variables). LC: laparoscopic cholecystectomy; SSI: surgical site infection; LOS: length of hospital stay; IQR: interquartile range; N: total number of patients; n: number of patients

Outcomes	Completed LC (N=223)	Converted (N=49)	p-Value
Any complication, n (%)	25 (11.2)	15 (30.6)	0.001
SSI, n (%)	12 (5.4)	8 (16.3)	0.014
Bile leak, n (%)	3 (1.3)	2 (4.1)	0.222
Bile duct injury, n (%)	0	0	-
Intra-abdominal collection, n (%)	2 (0.9)	2 (4.1)	0.15
Ileus, n (%)	7 (3.1)	4 (8.2)	0.116
Pulmonary complications, n (%)	5 (2.2)	2 (4.1)	0.613
LOS (days), median (IQR)	4 (3-5)	7 (6-9)	<0.001
30-day readmission, n (%)	5 (2.2)	2 (4.1)	0.613
Mortality, n (%)	0	1 (2.0)	0.18

According to the Clavien-Dindo classification, most complications were low grade (Table 4). In the completed LC group, 22/25 (88.0%) complications were classified as grade I-II, and 3/25 (12.0%) were classified as grade III. In the converted group, 12/15 (80.0%) were grade I-II, 2/15 (13.3%) were grade III, and 1/15 (6.7%) was grade V (mortality). No grade IV complications were observed in either group.

**Table 4:** Postoperative complications classified by Clavien-Dindo grade. LC: laparoscopic cholecystectomy; N: total number of patients; n: number of patients

Clavien-Dindo grade	Completed LC (complications n=25)	Converted (complications n=15)	Total complications (N=40)
Grade I-II	22 (88.0%)	12 (80.0%)	34 (85.0%)
Grade III	3 (12.0%)	2 (13.3%)	5 (12.5%)
Grade IV	0 (0.0%)	0 (0.0%)	0 (0.0%)
Grade V	0 (0.0%)	1 (6.7%)	1 (2.5%)
Total	25 (100%)	15 (100%)	40 (100%)

**Figure 2:**
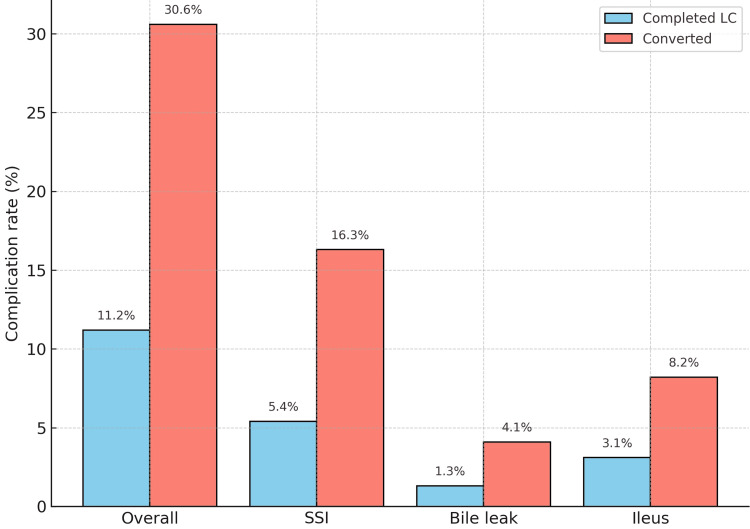
Postoperative complication rates in completed laparoscopic cholecystectomy vs. converted cases. Only major differentiating complications (overall, SSI, bile leak, and ileus) are displayed; less frequent complications are detailed in Table 3. LC: laparoscopic cholecystectomy; SSI: surgical site infection

As illustrated in Figure 2, overall complications were significantly higher in converted cases compared with completed LC (15/49, 30.6% vs. 25/223, 11.2%). Surgical site infection was also more frequent after conversion.

Table 5 summarizes outcomes in completed LC stratified by drain use. Complications occurred in 4/30 (13.3%) of patients with drains compared with 21/193 (10.8%) without drains (p=0.76). SSI was reported in 2/30 (6.7%) vs. 10/193 (5.2%) (p=0.66). The median hospital stay was longer in patients with drains (five vs. four days, p=0.03).

**Table 5:** Outcomes in completed laparoscopic cholecystectomy: drain vs. no drain. P-values derived from Mann-Whitney U test (LOS) and Fisher’s exact test (categorical variables). SSI: surgical site infection; LOS: length of hospital stay; IQR: interquartile range; N: total number of patients; n: number of patients

Outcomes	Drain (N=30)	No drain (N=193)	p-Value
Any complication, n (%)	4 (13.3)	21 (10.8)	0.755
SSI, n (%)	2 (6.7)	10 (5.2)	0.66
LOS (days), median (IQR)	5 (4-6)	4 (3-5)	0.03

Table 6 summarizes outcomes in converted cases stratified by drain use. Complications were observed in 10/32 (31.3%) with drains compared with 5/17 (28.6%) without drains (p=1.00). SSI occurred in 5/32 (15.6%) vs. 3/17 (17.6%) (p=1.00). Median hospital stay was longer in patients with drains (eight vs. six days, p=0.04).

**Table 6:** Outcomes in converted cases: drain vs. no drain. P-values derived from Mann-Whitney U test (LOS) and Fisher’s exact test (categorical variables). SSI: surgical site infection; LOS: length of hospital stay; IQR: interquartile range; N: total number of patients; n: number of patients

Outcomes	Drain (N=32)	No drain (N=17)	p-Value
Any complication, n (%)	10 (31.3)	5 (28.6)	1.00
SSI, n (%)	5 (15.6)	3 (17.6)	1.00
LOS (days), median (IQR)	8 (6-10)	6 (5-7)	0.04

## Discussion

In this retrospective cohort study of 272 patients undergoing laparoscopic cholecystectomy (LC), we found that the majority of procedures (82%) were successfully completed laparoscopically, while 18% required conversion to open cholecystectomy. Patients undergoing conversion had significantly higher complication rates, longer operative times, and increased length of hospital stay compared with those who had completed LC. Drains were used selectively at the discretion of the operating surgeon. Their use did not reduce postoperative morbidity but was consistently associated with prolonged hospitalization. These findings reinforce the established role of LC as the standard of care for gallstone disease, highlight the clinical implications of conversion, and add to the growing evidence against routine drain use in cholecystectomy.

Our overall conversion rate of 18% is somewhat higher than the 2-15% typically reported in international series [6,7]. This difference may be explained by the high proportion of patients presenting with acute or complicated gallbladder disease at our center, as well as the variable surgical experience of operating teams. Rosen et al. identified acute inflammation, adhesions, and distorted anatomy as major predictors of conversion [6], while Bingener et al. demonstrated that elderly patients are more likely to require conversion [7]. In our cohort, emergency procedures and hostile intraoperative anatomy were significantly more common in the converted group, which likely explains the higher conversion rate. Importantly, conversion should not be regarded as a complication but rather as a protective intraoperative decision to ensure safety. Gupta and Jain have emphasized the importance of a “culture of safety” in LC, advocating for liberal conversion when the critical view of safety cannot be obtained [5]. From this perspective, our conversion rate may represent a cautious but appropriate surgical approach in a setting where severe inflammation is frequently encountered.

The differences in postoperative outcomes between completed LC and converted cases observed in our study are consistent with prior reports. The overall complication rate after completed LC was 11.2%, which aligns with the 5-12% range described in large multicenter series [3,4]. In contrast, conversion was associated with nearly a threefold increase in morbidity (30.6%). Surgical site infection (SSI) was the most frequent complication in both groups, but its incidence was markedly higher in converted cases (16.3% vs. 5.4%). Bile leak and intra-abdominal collections were rare but occurred more often in conversions. Peters et al. similarly reported higher rates of SSI, pulmonary complications, and prolonged operative time in converted cases [8]. Nilsson et al., in a population-based Swedish study, also confirmed that open and converted procedures are linked to increased morbidity, longer recovery, and higher healthcare costs compared with LC [15]. These findings underscore the clinical and economic burden imposed by conversion, even though it remains an essential safety measure.

Length of hospital stay is another important outcome, especially in low- and middle-income settings. Patients with completed LC in our series had a median LOS of four days compared with seven days in converted cases. Coccolini et al., in their meta-analysis, showed that LC consistently resulted in shorter hospital stays compared with open surgery [4]. While our study did not analyze elective open cholecystectomy, the outcomes in converted patients approximate those of open procedures, reflecting their increased invasiveness. This reinforces the advantage of LC in terms of recovery and resource utilization, while also acknowledging the unavoidable burden when conversion is necessary.

The role of drains in cholecystectomy remains controversial. Drains have traditionally been used to prevent bile collections and to detect bleeding [9]. However, robust evidence now indicates that routine drainage offers no benefit. In our study, drains were placed in 13.5% of completed LC and 65.3% of converted cases. Complication rates did not differ significantly between drain and no-drain groups, but hospital stay was consistently longer when drains were used. These findings mirror those reported by Gurusamy et al. in a Cochrane review, which found no reduction in postoperative morbidity but higher pain and delayed discharge with drains [10]. Picchio et al., in a meta-analysis, similarly concluded that drains do not reduce SSI, bile leak, or intra-abdominal abscess but do prolong LOS [11]. Our results also align with Indian data from Sharma and Mittal, who demonstrated that routine drains after uncomplicated LC were unnecessary and led to longer hospitalization [13]. More recently, a study by Kim et al. showed that even in acute cholecystitis, prophylactic drains did not reduce morbidity [14]. These results are in accordance with international recommendations, including the WSES guidelines, which discourage routine drainage and advocate selective use only in technically challenging cases [12]. Together, these data strengthen the argument that drains should not be placed routinely after LC or conversion.

The consistency of our findings with international evidence reinforces their validity. Keus et al., in a Cochrane review, confirmed that LC is superior to open cholecystectomy for symptomatic gallstone disease [3], while Coccolini et al. demonstrated similar benefits in the setting of acute cholecystitis [4]. Although our study compared completed and converted LC rather than elective open procedures, the contrast in outcomes is parallel as follows: LC yields better clinical results and shorter recovery, while conversion is associated with greater morbidity. The additional analysis of drain use adds further evidence to the international consensus that drains should not be routine.

This study has limitations. As a retrospective, single-center analysis, it is subject to selection and documentation bias and may limit generalizability. Drain placement was non-random and selective at the surgeon’s discretion; therefore, comparisons between drain and no-drain groups are susceptible to selection bias, and small subgroup sizes, particularly within the converted cohort, reduced statistical power and precluded multivariable adjustment. We also did not assess long-term outcomes, such as incisional hernia or quality of life. Despite these constraints, the study presents contemporary real-world data from an Indian tertiary care setting on conversion outcomes and the effects of selective drainage in cholecystectomy.

Future research should focus on prospective multicenter studies with larger sample sizes to validate these findings and to better define risk factors for conversion in diverse patient populations. Randomized trials specifically addressing drain use in converted cases are lacking and would be valuable to confirm whether selective drainage can ever confer benefit. Additionally, the development and validation of predictive models to stratify patients at high risk of conversion could help optimize surgical planning, resource allocation, and patient counseling.

## Conclusions

Laparoscopic cholecystectomy was successfully completed in the majority of patients and remained the preferred approach for gallstone disease, with lower morbidity and shorter hospitalization compared with cases that required conversion. Conversion, though sometimes unavoidable, was associated with higher complication rates and longer recovery, underscoring the importance of careful preoperative risk assessment and intraoperative decision making. Although drains were selectively placed rather than used routinely, their presence did not reduce postoperative complications in either completed or converted cases, and was associated with prolonged hospital stay. These findings support selective rather than routine drain use and reinforce the role of laparoscopic cholecystectomy as the standard of care, in line with international recommendations such as the WSES guidelines.
